# A context-based approach to identify the most likely mapping for RNA-seq experiments

**DOI:** 10.1186/1471-2105-13-S6-S9

**Published:** 2012-04-19

**Authors:** Thomas Bonfert, Gergely Csaba, Ralf Zimmer, Caroline C Friedel

**Affiliations:** 1 Institute for Informatics, Ludwig-Maximilians-University Munich, Amalienstr. 17, 80333 Munich, Germany

## Abstract

**Background:**

Sequencing of mRNA (RNA-seq) by next generation sequencing technologies is widely used for analyzing the transcriptomic state of a cell. Here, one of the main challenges is the mapping of a sequenced read to its transcriptomic origin. As a simple alignment to the genome will fail to identify reads crossing splice junctions and a transcriptome alignment will miss novel splice sites, several approaches have been developed for this purpose. Most of these approaches have two drawbacks. First, each read is assigned to a location independent on whether the corresponding gene is expressed or not, i.e. information from other reads is not taken into account. Second, in case of multiple possible mappings, the mapping with the fewest mismatches is usually chosen which may lead to wrong assignments due to sequencing errors.

**Results:**

To address these problems, we developed ContextMap which efficiently uses information on the context of a read, i.e. reads mapping to the same expressed region. The context information is used to resolve possible ambiguities and, thus, a much larger degree of ambiguities can be allowed in the initial stage in order to detect all possible candidate positions. Although ContextMap can be used as a stand-alone version using either a genome or transcriptome as input, the version presented in this article is focused on refining initial mappings provided by other mapping algorithms. Evaluation results on simulated sequencing reads showed that the application of ContextMap to either TopHat or MapSplice mappings improved the mapping accuracy of both initial mappings considerably.

**Conclusions:**

In this article, we show that the context of reads mapping to nearby locations provides valuable information for identifying the best unique mapping for a read. Using our method, mappings provided by other state-of-the-art methods can be refined and alignment accuracy can be further improved.

**Availability:**

http://www.bio.ifi.lmu.de/ContextMap.

## Background

Deep sequencing of mRNA using next-generation sequencing technologies (RNA-seq) offers novel opportunities to profile and quantify whole transcriptomes. The nucleotide-level resolution of RNA-Seq experiments provides new insights for researchers into the complexity of alternative splicing and isoform expression [1-5]. One major challenge in RNA-seq is the identification (mapping) of the origin of each sequenced read, i.e. which part of which transcript it corresponds to. As this requires the alignment of a huge number (in the order of millions) of relatively short sequence reads against reference sequences, such as e.g. a genome or transcriptome, a number of specialized alignment algorithms have been developed. Here, algorithms based on the FM-index - a compressed, searchable suffix array-like structure [6] - have been most successful due to their reduced time and memory requirements. The most widely used of these algorithms is Bowtie [7].

The complex structure of a spliced transcriptome compared to the genome limits the applicability of simple alignment-based approaches for RNA-seq experiments. While an alignment against the genome can successfully map reads from an unspliced region of the transcriptome, such as individual exons or introns, reads from spliced regions are missed. To some degree, this problem can be addressed by an alignment against a known transcriptome or a database containing exons and all possible junctions between these exons [8,9]. However, in the first case, the sensitivity of this approach depends strongly on the completeness of the annotated transcriptome and novel splice junctions will be missed. In the second case, the number and complexity of potential junction reads increases dramatically with increasing read length resulting in forbiddingly large databases.

As a consequence, a large number of more sophisticated bioinformatics approaches have been developed for the identification of junction reads. One of the first tools was TopHat [10] which is able to discover splice sites without the use of a transcriptomic annotation. In a first phase, reads are aligned to a reference genome using Bowtie and the mapped reads are then assembled to so-called islands. Subsequently, potential splice sites are annotated based on canonical splice signals and reads spanning these splice sites are identified. In contrast, two more recently published methods, RUM [11] and RNASEQR [12], start with read alignments to both the reference transcriptome and genome. Novel splice junctions are then identified by aligning unmapped reads to the genome using BLAT [13] which determines gapped local alignments. A gapped alignment only becomes feasible here because it is applied to the much smaller number of unaligned reads.

Thus, all of these approaches rely heavily on additional information such as canonical splice sites or reference transcriptome annotations. A more sophisticated approach which is independent of the availability of such additional information is MapSplice [14]. The general idea of MapSplice is that it first generates candidate alignments for each read based on alignments of short fragments of the read against the genome. Splice junctions are then predicted based on all candidate alignments for different reads containing the respective splice site. Thus, in contrast to previous approaches MapSplice takes into account the context of a splice site, i.e. how many reads support the predicted splice site.

While this approach is interesting, there are still a few limitations. first, the context of other read mappings is taken only into account for potential splice sites. For reads mapping to an unspliced region, the context is ignored. Second, only spliced read alignments are considered but not unspliced reads within this range. In particular, unspliced reads ending or starting at the potential splice junction might lend additional support to the splice site. Third, for each fragment only the alignments with the minimum number of mismatches is considered ignoring the possibility that the alignment to the correct origin may have more mismatches due to sequencing errors. Finally, although non-unique alignments are outputted by MapSplice, no effort is made to resolve them. While some downstream analysis methods have been developed which can deal with this uncertainty (e.g. for the estimation of transcript levels [15-17]), often these multi-maps are simply discarded and, thus, lost for the analysis.

In this article, we present a novel method (ContextMap) which extends the idea of using the context of other read alignments for identifying the correct position for each read. This method is more general than MapSplice as it uses the context not only for splice junctions but also non-spliced read alignments and always uses both spliced and unspliced read alignments. The general idea of the approach is not to assign each read to the position where it can be aligned with the fewest mismatches, but to the position where it fits best in the context with its surrounding and all other reads. For this purpose, we allow a high degree of ambiguity during the mapping stage which is eventually resolved in the final mapping. Thus, ContextMap is robust against biases caused by sequencing errors and may also be used for correction of sequencing errors.

ContextMap can be used in stand-alone and incremental mode. The incremental mode starts with an initial alignment of a mapping method of choice and refines this initial mapping. In the stand-alone mode, it obtains this initial mapping by first aligning the reads to the genome, the transcriptome or both. In the implementation we present here, ContextMap uses as initial context the unique mapping identified by MapSplice or TopHat. Evaluation on simulated reads for human and mouse showed a significant improvement in mapping quality of the ContextMap refinements compared to both MapSplice and TopHat. This highlights the importance of using the read context for obtaining the final mapping.

## Methods

### Outline

The central concept of ContextMap is a *contex*t of reads. A context is a set of reads which all originate from the same expressed stretch of the genome. In general, such a context corresponds to an individual gene but may also correspond to a few overlapping or closely located genes. At any step of ContextMap, only reads within the same context will be considered for assigning a position to the read within the *specific *context considered. At least initially, however, reads may be assigned to different contexts and in this case are assigned a position for each context. This ambiguous assignment of reads to contexts is eventually resolved in the final step of ContextMap.

**Figure 1:**
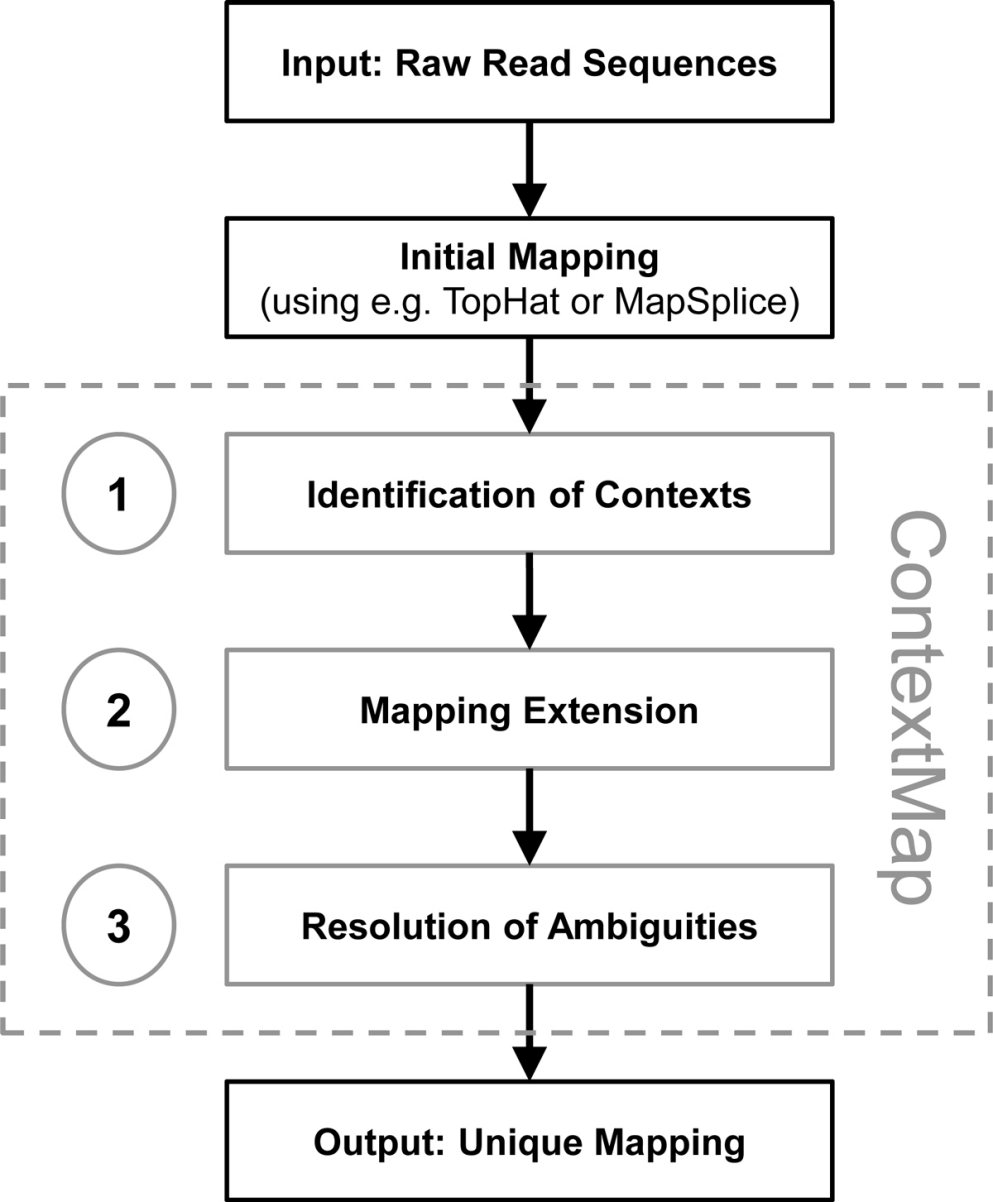
**Outline of the ContextMap**. ContextMap takes as input initial alignments provided either by other mapping tools such as e.g. TopHat or MapSplice or by a genome or transcriptome alignment. As output unique locations are provided for all reads that can be mapped. ContextMap involves three steps which are explained in more detail in Figures 2-3: (1) Identification of contexts (see Figure 2A); (2) Extension of initial alignments (Figure 2B-C); (3) Resolution of ambiguities (Figure 3).

ContextMap then consists of the following three steps (see Figure 1): (1) the definition of initial contexts using a preliminary mapping of reads; (2) the extension of the preliminary mapping by re-aligning reads within their respective contexts; (3) the resolution of ambiguous mappings both within and between contexts to obtain a final unique mapping. Here, the advantage of using contexts is that we can allow a much larger degree of ambiguity during the second step, as ambiguities are limited to individual contexts and not the whole genome and the contexts allow us to resolve the ambiguities successfully. In the following, the individual steps are explained in more detail.

### Identification of contexts

**Figure 2:**
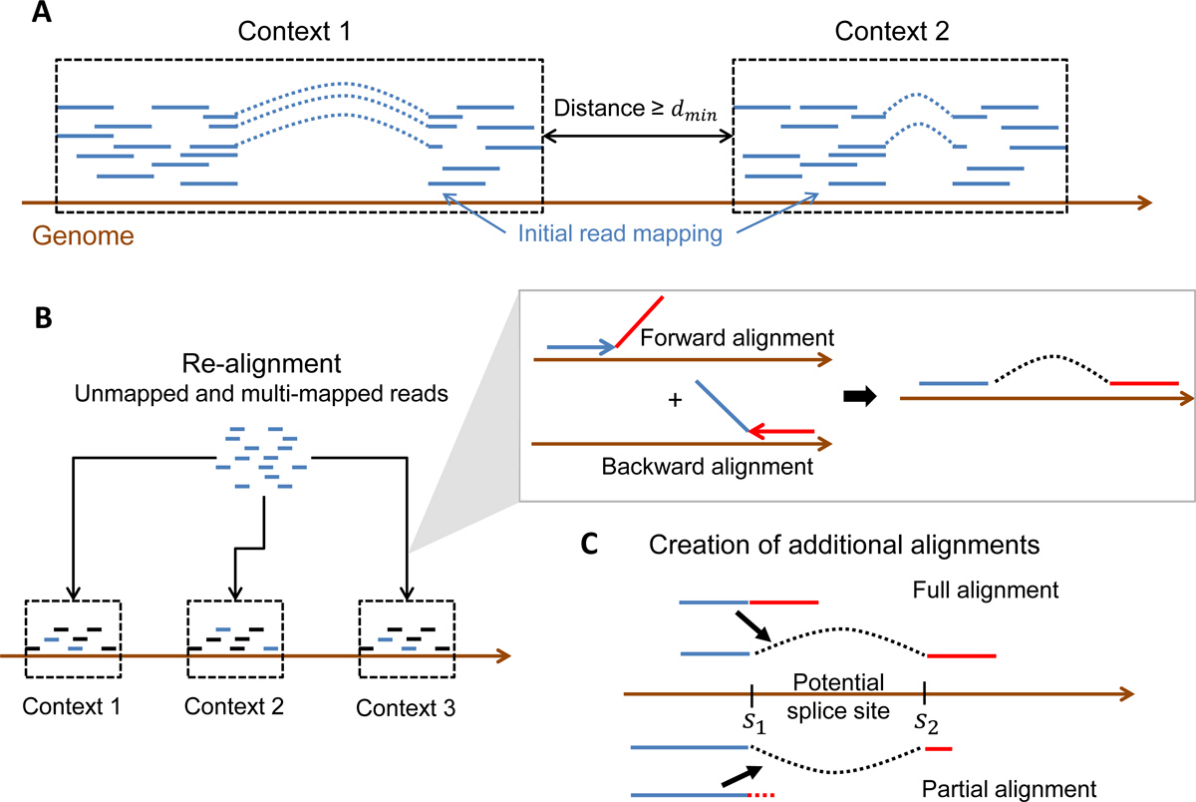
**Definition of contexts and extension of alignments**. A) A context is defined as a set of reads with overlapping mapping locations such that the minimum distance between any pair of reads from different contexts is *d_min_*. B) The initial alignments used as input to ContextMap are extended by re-aligning unmapped or multi-mapped reads to each context using a forward alignment from the read start towards its end and a backward alignment from the read end towards its start. Forward and backward alignments are combined to a full or split read alignment if the maximum mismatch criterion is met. (C) All alignments obtained at this step provide a list of potential splice sites. Additional alignments respecting the maximum mismatch criterion are then created for full and partial read alignments based on these potential splice sites. Accordingly, for any read aligning up to the first position *s*_1 _of a splice site it is investigated if the rest of the read can be aligned starting at the second position *s*_2 _of the splice such that only a maximum number of mismatches are created. These additional alignments are then added to the set of candidate alignments.

The initial step of ContextMap requires the definition of contexts, i.e. the definition of reads from the same expressed region of the genome (Figure 2A). Here, the preliminary context does not have to be fully correct as it only roughly defines putatively expressed regions. In particular, the precise alignment of the reads is of lesser importance, as reads will be realigned in a subsequent step to identify a larger number of candidate alignments.

There are several ways in which a context can be identified. The solution we implemented for this study is to use the alignment of other mapping algorithms such as MapSplice or TopHat which we aim to improve upon. In this case, all unique mappings determined by the used method are included. Alternatively, ContextMap can identify contexts itself by an initial alignment of reads to the genome using the forward and backward approach described in the following section. Potential splice junction reads are then identified by searching for easily detectable (balanced) splits. Instead of an alignment to the genome, an alignment to the transcriptome may also be used to identify the initial mapping.

The contexts are then defined based on the genomic distance between aligned reads. Reads with a maximum distance of *d_min _*between start or end positions are collected into the same context. Accordingly, the minimum distance between contexts is *d_min_*. Note that contexts may contain regions without mapped reads larger than *d_min _*if these regions are contained within the spliced part of a read, i.e. an intron. The distance threshold can be adjusted if smaller or larger contexts are desired. In this study, *d_min _*was set to 10 kb. Alternatively, gene annotations may be used to define contexts, which then limits the mapping to known genes.

### Extension of candidate mappings

The first part of this step is a re-alignment of previously unmapped or non-uniquely mapped reads to each context (Figure 2B). In the following, alignents containing no splice site are denoted as full, whereas alignments to splice sites are denoted as split alignments/reads. In this step, all full and split alignments fulfilling a maximum mismatch criterion are generated using Bowtie in the following way. For each context, both a forward and backward Bowtie index is created. Using these indices, reads are then aligned to the contexts both in forward direction starting from the read start and in reverse direction starting from the read end. For both alignments a seed of 40% of the read (but at most 40 bp) is used allowing a maximum number of 1 mismatch in the seed region. Again, these are parameters which can be adjusted by the user depending on read length and expected error rates. Forward and backward alignments are then combined if the maximum number of mismatches in the alignment does not exceed the predefined threshold.

These alignments as well as the initial alignments provide a set of potential splice sites for each context. Each splice site is characterized by two sequence positions (*s*_1_, *s*_2_) such that any read covering the splice site will first align on the genome upstream of and up to *s*_1 _and then continue to align at *s*_2 _and downstream of this position. Using the potential splice sites for each context, additional alignments are generated in the following way (Figure 2C). For reads which have a full alignment crossing either *s*_1 _or *s*_2 _for a splice site, any alignment respecting the splice site and fulfilling the maximum mismatch criterion is generated. The same is done for partial read alignments in which the read could be aligned except for a small fragment at the start or the end, but the fragment was to small to obtain a meaningful alignment. As the number of potential splice sites suggested by alignments of other reads is limited, all of these splice sites can be tested whether they allow an alignment of the unaligned fragment fulfilling the maximum mismatch criterion.

## Resolution of ambiguities

### Redundancy filtering

**Figure 3:**
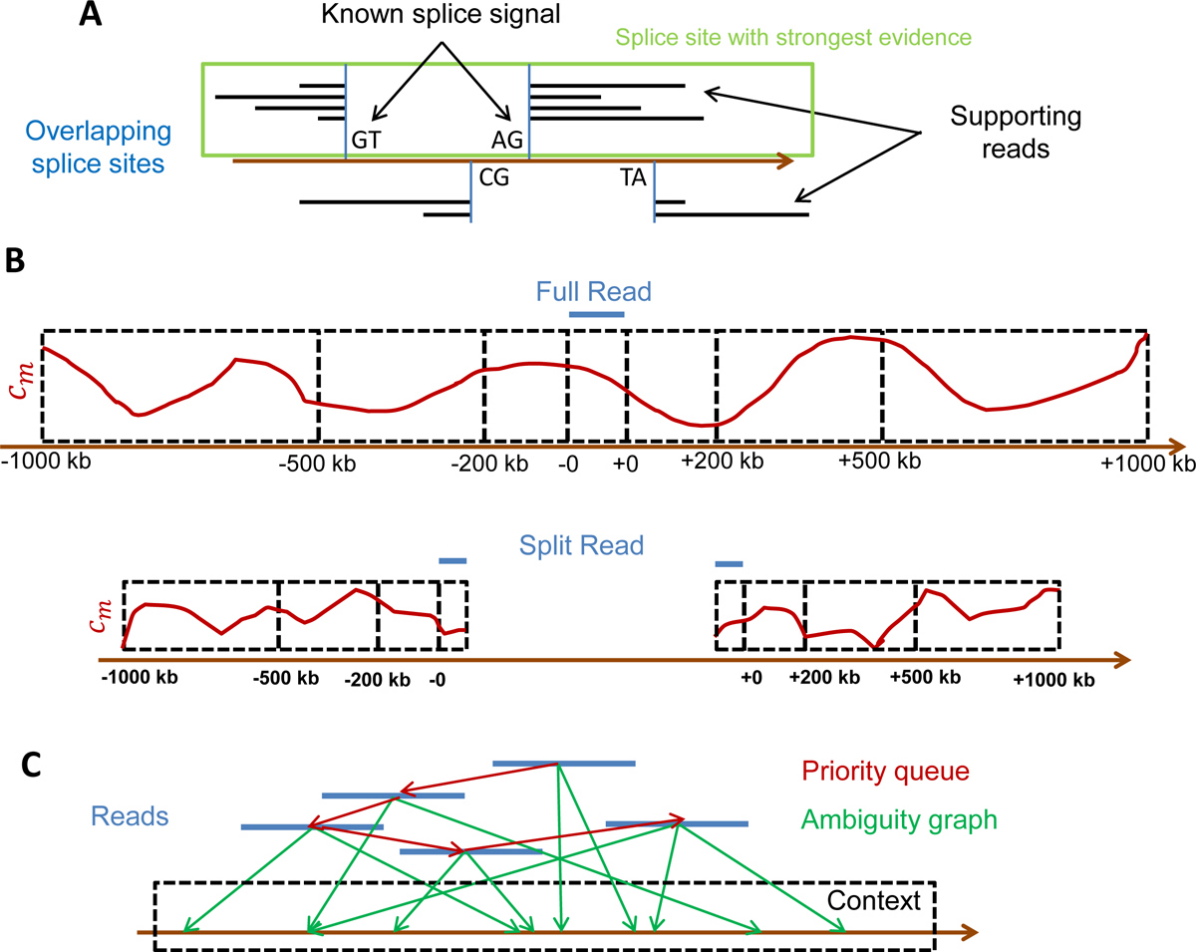
**Resolution of ambiguities**. A) Among overlapping splice sites, the one with the best evidence is chosen. Here, two potential splice sites are shown (blue) with distance less than the read length. Evidence is provided by reads supporting the splice site (black lines) as well as known splice signals. In this example, the left-most splice site has more reads supporting it (4 compared for 2 for the right-most splice site) as well as the canonical splice signal GTAG. Thus, this splice site is chosen and the other one is discarded. B) For ambiguous mappings of the same read within a context, coverage scores are calculated based on the number of reads mapping per position (*c_m_*, red curves) within the read alignment region and increasingly large windows around the read alignment. Here, -0 indicates the aligned position of the read start and +0 the position of the read end. A negative sign indicates positions upstream of the alignment and a positive sign positions downstream. Region 1 includes the positions from -200 to -0 and +0 to +200 bp, region 2 the positions from -500 to -200 and +200 to +500 bp, and so on. For split alignments only aligned read positions as well as positions upstream of the read start and downstream of the read end are included in the coverage calculation. (C) For the final ambiguity resolution for each context, reads (blue) are connected in a graph (green) to all possible locations in the context they can be mapped to. Furthermore, a priority queue (red) is created in which the reads are sorted according to the difference in coverage scores between the best and second-best alignment. Iteratively, the read with the currently largest difference is removed from the queue, its position is fixed for the context and coverage scores in the queue are updated.

Having generated a large number of ambiguous mappings for each read and context which respect the maximum mismatch criterion, the next step is then to resolve the ambiguities within each context before addressing the ambiguities between contexts (Figure 3). To not unnecessarily bias this step by reads which will be later removed anyway as they are assigned to a different context, we use an additional parameter which specifies the maximum mismatch difierence (*δ_mm_*) for a read in any context to the best-matching context. For this purpose, we calculate for each read and each context the minimum number of mismatches in any of the candidate alignments. If the best match of a read in a context *c *requires >*δ_mm _*mismatches more than in the best-matching context, the read is removed from *c*.

To further simplify the resolution process, full and split mappings with either the same start or end position are merged for this step. The final configuration for these reads (full or split) is then determined at the end of this step. In addition, we identify the best supported splice site among any pair of overlapping splice sites (Figure 3A). Two splice sites (*s*_11_, *s*_12_) and (*s*_21_, *s*_22_) are overlapping if |*s*_11 _- *s*_21_| < read length and |*s*_12 _*- s*_22_| < read length. The idea behind this approach is to eliminate splice sites which are too close to each other and, thus, make no biological sense or are suggested by alternative split alignments of the same read. For this purpose, we calculate the number of supporting reads (full, split or partial) for each splice site and the corresponding mismatch cost and check which of the splice sites has a known splice signal. In the following, let *n_i _*be the number of reads with *i *mismatches supporting the splice site and *m *the maximum number of mismatches allowed. Furthermore, *λ *= 2 if the splice site contains a known splice signal and *λ *= 1 otherwise. The evidence score is then calculated as

(1) $$\mathrm{evidence}\;=\;\lambda \;\cdot \;\overset{m}{\underset{i=0}{\sum }}\left(0.3^{i}\;\cdot n_{i}\right)\;,$$

Thus, the score is the weighted sum of the number of reads with the weight decreasing exponentially with the number of mismatches. For each set of pairwise overlapping splice sites, the one with the largest evidence score is then chosen.

### Calculation of read coverage scores

To obtain the unique mapping of each read within each context, we calculate a coverage score for this read in the following way (Figure 3B). First we calculate for each position the number of reads mapping to this position (*c_m_*). If there are ambiguous mappings for a read, it is counted for all positions in any of the ambiguous mappings. We then define 4 regions within and around the mapped read. Region 1 contains the positions the read is aligned to. Region 2 contains all positions 200 bp either upstream of the read start or downstream of the read end. Region 3 corresponds to the positions > 200 but ≤ 500 bp from read start or end and, finally, Region 4 to positions > 500 but ≤ 1000 bp from read start or end. The score for each region, *score_i_*, is then defined as the maximum *c_m _*within the corresponding region *i*. Region sizes and numbers may be adjusted depending on the user needs.

Finally, the coverage of a read is calculated as

(2) $$\;\mathrm{coverage}\;=\;\;\;\overset{4}{\underset{i\;=\;1}{\sum }}2^{4-i}\;\cdot \;\left\lfloor \text{ln}\left(\mathrm{scor}e_{i}\right)\right\rfloor \;.$$

This calculation involves three aspects. First, the definition of regions for which the maximum *c_m _*is calculated allows to take into account reads mapping very far from the actual read alignment without assigning too much weight to distant reads. The larger the region, the less weight is given to reads falling in this region. Second, the individual region scores are additionally weighted depending on the distance from the actual read alignment. Reads overlapping the alignment region have the largest weight and each region has a weight twice as high as the next more distant region. Finally, instead of the actual read counts logarithms of the counts are used, reflecting their order of magnitude. In this way, this coverage definition is related to the geometric mean of the scores which is less biased by large outliers than the arithmetic mean.

### Priority queue and ambiguity graph

The ambiguous reads are then put into a priority queue sorted by the difference in coverage score between the best and second-best ambiguous mapping (Figure 3C). Furthermore, a graph structure is created for each context in which each ambiguous read is connected with the positions in this context to which it maps (ambiguity graph). Thus, the ambiguity graph provides the information on which coverage scores have to be updated if a read is assigned to a unique position.

We then iteratively remove the entry with the highest score from the queue, i.e. the entry for which the ambiguity resolution is the most straightforward. Subsequently, we update the coverage scores based on the dependencies identified by the graph structure and then update the keys in the queue. Finally, when the queue is empty, all ambiguous reads have been assigned a unique location within the respective context. For a speed-up, the costly coverage updates were omitted in this study and ambiguities are resolved only based on the highest (initial) coverage score.

In the last part of this step, the assignment for the merged full and split mappings with either same read start or end is resolved. For this purpose, coverage scores are again calculated but this time only for the positions which are differently mapped by the alignment and the positions upstream of the read starts, in case of differential start points, or downstream of the read end, in case of differential end points. The alignment with the largest coverage is then chosen.

To resolve the between-context ambiguities, we basically perform the same steps as for each individual context by calculating coverage scores for each context and using a priority queue based on the coverage differences and a global dependency graph between reads and contexts. Finally, resolved read mappings are outputted if the relevant region suggests a real expression. Currently, we predict reads with at least 100 other reads mapped in a region within 1000 bp up- or downstream of the read. These are again user-defined thresholds.

## Results

### Simulation of RNA-seq data sets

**Figure 4:**
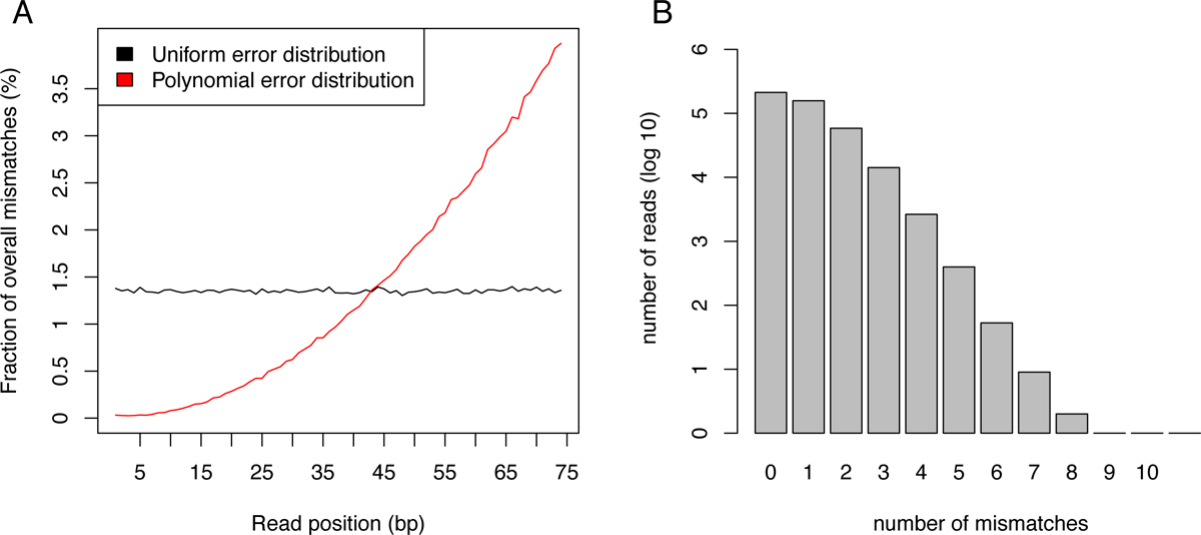
**Mismatch distributions**. (A) Distribution of mismatches along the read length are shown for the uniform and polynomial mismatch distributions. (B) Distributions of the number of mismatches per read are shown for the human data set with 1% error rate.

All approaches were evaluated on simulated human (hg19) and mouse (mm9) reads. For the human reads a 74 bp single end RNA-Seq data set was generated with the FLUX simulator [18]. Since FLUX was too slow for simulating reads for the whole human genome we restricted the simulation to chromosome 1 and obtained a final set of 446, 398 reads. We then randomly introduced sequencing errors into this original read set to obtain two data sets with 1% and 2% uniformly distributed errors, respectively (Figure 4). Since sequencing quality generally deteriorates towards the end of the read, we also simulated reads with error probability increasing along the read length (Figure 4A). In this case, the overall error rate was again fixed at 1% or 2%, respectively, but the position of the error in a read was drawn from a polynomial distribution with cumulative distribution function $F\left(x\right)\;=\;\frac{1}{l^{3}}x^{3}$, where l is the read length.

For the mouse read data, we used an evaluation set published by Grant *et al. *in the RUM algorithm [11], restricted to reads mapping to Ensembl transcripts. It contains 17,301,982 single end 100 bp reads. In the original RUM simulation model, two types of errors were simulated: random base and tail errors. Base errors were uniformly distributed across the whole read and tail errors only in the tail of a random fraction of the reads. For our purposes, we used the first test set with a base error of 0.5% and no additional tail error. The second test set with an additional tail error of 50% in the last 10 bases of 25% of reads was not used. In this case, trimming of reads by the last 10 bases would always result in a significantly improved performance. To test the performance for higher error rates, we also introduced sequencing errors in the mouse data set to obtain two sets with uniformly distributed 1% and 2% error rate, respectively.

The objective behind using two different error rates each for each data set was to have both a relatively easy data set with few sequencing errors as well as a more challenging set with a larger error rate. In the first case, we expect little reduction of the performance due to error rates. In the second case, a large influence is expected.

### Baseline mapping algorithms

To show that our approach is able to further improve the results of widely used mapping and junction discovery tools, we applied ContextMap to mappings which were produced with MapSplice and TopHat. Both of them are popular programs that are able to map RNA-Seq reads to a reference genome without using a transcriptome annotation. TopHat mappings were obtained allowing at most 2 mismatches per segment and read. For MapSplice we used default parameter settings except for the spliced mismatch parameter. For this we used 4 as otherwise MapSplice performed poorly for the data sets with a larger error rate. ContextMap was then applied both on the TopHat and MapSplice mappings.

### Read mapping accuracy

To evaluate the accuracy of a mapping, we calculated the accuracy of read mapping, i.e. the fraction of reads aligned to the correct positions. This was done separately for reads which were simulated as complete reads as well as for reads spanning a splice junction. A uniquely mapped read is counted as an *exact *true positive (TP) if it was mapped correctly at every base. In case a read was either an exact match or at least the start or the end position on the genome was correctly predicted, we counted it as a *relaxed *true positive. Reads not (uniquely) mapped or mapped to wrong positions were treated as false negatives (FN). Accuracy was then calculated as

(3) $$\mathrm{Accuracy}\;=\;\frac{\mathrm{TP}}{\mathrm{TP}\;+\;\mathrm{FN}}.$$

Evaluation results are shown in Tables 1 and 2 for the human and mouse data sets, respectively. As expected, all approaches performed best for complete read mappings and performance deteriorated considerably for the junction read mappings. However, if also we include the cases in which at least either the start or the end position of the read was predicted correctly, we see that in many cases at least part of the read could be mapped correctly.

**Table 1:** Evaluation results on human data sets

Data set	Program	Complete Read Mapping		Junction Read Mapping	
			
		exact	relaxed	exact	relaxed
Human, 1%	TopHat	93.85	93.85	72.18	80.73
	ContextMap^1^	95.76	95.76	**73.12**	**86.21**
	MapSplice	78.81	89.64	70.16	78.78
	ContextMap^2^	**95.97**	**95.98**	73.07	86.05

Human, 2%	TopHat	90.30	90.30	70.38	78.05
	ContextMap^1^	**95.51**	**95.52**	**71.53**	**83.78**
	MapSplice	53.92	81.45	58.73	68.14
	ContextMap^2^	95.45	95.48	64.13	78.68

Accuracy of read mapping is reported for the two human data sets with 1% and 2% error rate, respectively. Here, ContextMap^1 ^denotes the refinement of TopHat initial mappings by ContextMap, ContextMap^2 ^the refinement of MapSplice initial mappings.

**Table 2:** Evaluation results on mouse data sets

Data set	Program	Complete Read Mapping		Junction Read Mapping	
			
		Exact	relaxed	exact	relaxed
Mouse, 1%	TopHat	94.43	94.43	80.10	86.21
	ContextMap^1^	**97.03**	**97.05**	82.95	91.62
	MapSplice	95.21	95.22	84.49	89.12
	ContextMap^2^	96.97	96.99	**87.77**	**94.91**

Mouse, 2%	TopHat	88.87	88.88	77.44	82.87
	ContextMap^1^	95.82	95.87	80.45	88.52
	MapSplice	92.89	92.89	77.89	81.95
	ContextMap^2^	**96.31**	**96.36**	**82.87**	**90.57**

Accuracy of read mapping is reported for the two mouse data sets with 1% and 2% error rate, respectively. Notation is the same as in Table 1.

Our results clearly show that for both MapSplice and TopHat, ContextMap could improve significantly upon the predictive performance of the original mapping. In almost all cases, both variants of ContextMap outperformed both MapSplice and TopHat and in all cases at least one variant performed best. Interestingly, although MapSplice performed significantly worse than TopHat on the human data sets, ContextMap on MapSplice outperformed ContextMap on TopHat for the complete reads on the 1% error rate set by a small margin and was only worse by a similar small margin on the 2% error rate set. This was particularly impressive as in these cases MapSplice was > 15 percentage points worse than TopHat on the complete reads. Although MapSplice is usually a good mapping algorithm, we found that determining the optimal parameter settings for data sets with high error rates is rather difficult. In this case, ContextMap provides a useful alternative to parameter tuning as predictions can be refined considerably even if the parameter settings for MapSplice are not optimal.

**Figure 5:**
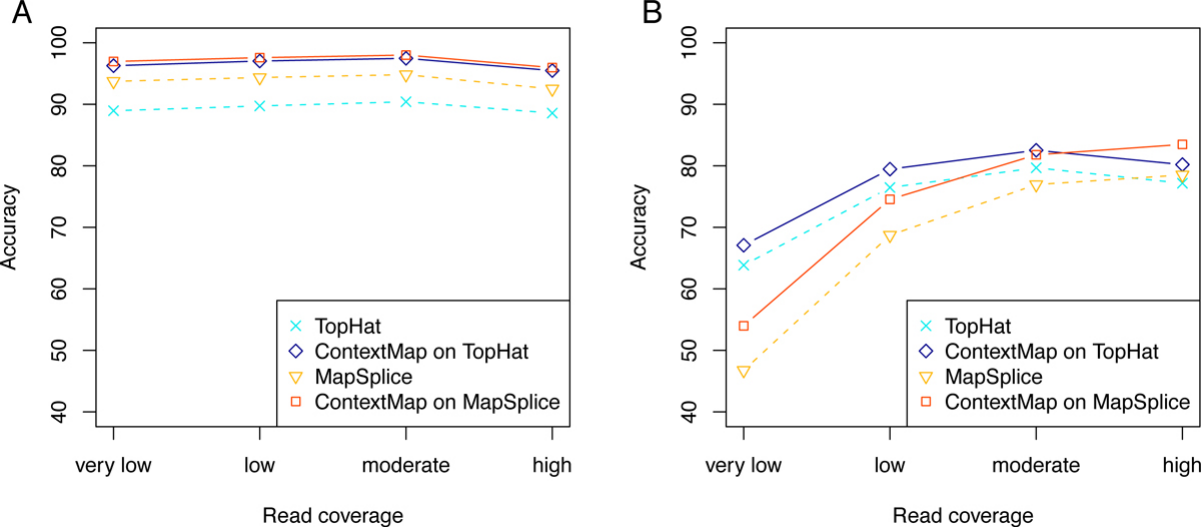
**Dependency of alignment accuracy on read coverage**. Alignment accuracy with respect to read coverage is shown for the mouse data set with 1% error rate: (A) complete reads and (B) junction reads. For this purpose, genes were binned into four approximately equal-sized groups based on the average read count per base and accuracy was calculated selectively for each group. For complete reads, no dependency was observed, whereas for junction reads, accuracy is strongly dependent on the read coverage.

To analyze whether alignment accuracy depended on read coverage, genes were partitioned into four approximately equal-sized groups based on the average number of reads per base. For each group, accuracy values were then calculated separately. This analysis showed that performance for complete reads without splice sites was mostly independent of read coverage (Figure 5A). In contrast, for junction reads, accuracy depended strongly on read coverage (Figure 5B). Generally, the higher the coverage was for a gene, the higher was the accuracy of the corresponding read alignments. Thus, read coverage is important for the identification of (novel) splice junctions, likely due to the much larger number of possible alignments, but not for alignment of reads originating from unspliced transcript regions. Nevertheless the relative ranking of the evaluated mapping approaches was generally only little influenced by read coverage. Interestingly, on the mouse 2% error data set (Figure 5B), ContextMap on MapSplice only outperformed ContextMap on TopHat for splice junctions in genes with highest read coverage. Since these genes contribute the largest set of reads to the overall read set, they mostly determined overall accuracy values and, accordingly, overall performance of ContextMap on MapSplice was also superior.

Interestingly, we found that alignment accuracy for both complete and junction reads depended little on the distribution of errors along the reads. Alignment accuracy on the human data set was almost identical no matter whether we used uniform or polynomial error distributions along the read length. The only method that suffered consistently from a non-uniform error distribution was TopHat with a decrease of around 4 percentage points for both types of reads on the 2% error data set with polynomial error distribution. Remarkably, however, accuracy of ContextMap on TopHat mappings was not reduced for the complete reads on this set despite the reduced quality of the initial mapping. Only accuracy for junction reads suffered but still was significantly higher than for TopHat and MapSplice, respectively.

Finally, we evaluated parameter sensitivity of ContextMap by running it with different seed lengths (values of 10, 20, 30, 40) and different minimum expression values for contexts (0-200 in steps of 20) on the human data sets. For both TopHat and MapSplice original mappings, we generally observed only minimal variation in accuracy with standard deviations of < 0.51 and < 0.21 percentage points for the seed (on complete reads) and minimum expression value parameters, respectively. The largest variation was observed on junction reads for the seed length parameter with a standard deviation of 1.2-2.9 percentage points. Remarkably, alignment accuracy was actually increased by using shorter seed lengths due to a larger number of junction reads correctly aligned. Unfortunately, it also resulted in a slight increase in the number of junction reads aligned incorrectly instead of not at all as in the case of longer seed lengths.

### Runtime

ContextMap increases the accuracy considerably compared to the baseline mapping programs. However, this comes at the cost of additional runtime as it requires running both the baseline mapping algorithms for the initial mapping as well as ContaxtMap for mapping refinements. Table 3 lists the runtime required for each step on 8 cores of identical machines with 48 G RAM. Here, several observations can be made. First, runtime of MapSplice was reduced by a factor of more than 50% compared to TopHat. Unfortunately, this reduced runtime also came at the cost of a significantly reduced accuracy. Second, the additional runtime required by ContextMap was very similar no matter which of the initial mappings was used. As a consequence, the relative runtime overhead created by ContextMap was much smaller for TopHat than for MapSplice. However, as the accuracy of MapSplice deteriorated considerably for the 2% error data sets, the additional runtime required by ContextMap payed off in a tremendous increase of accuracy in these cases. Finally, the combination of MapSplice with ContextMap required only little extra runtime compared to TopHat alone (7-14%) but outperformed TopHat substantially with the only exception of junction reads for the human 2% error rate set.

**Table 3:** Runtime comparisons

Data set	TopHat	ContextMap^1^	MapSplice	ContextMap^2^
Human, 1%	15.9	11.6	6.7	11.4
Human, 2%	17.8	11.8	7.9	11.8

Mouse, 1%	400.6	257.2	119.1	309.2
Mouse, 2%	578.9	401.2	176.7	460.3

Elapsed user times in minutes on the evaluated data sets is shown for each program. Every method was run using 8 cores on identical machines with 48 GB RAM. Here, runtime for ContextMap^1 ^and ContextMap^2 ^is the time required for the refinement step of ContextMap on the initial mappings provided by TopHat and MapSplice, respectively. It does not include the time required to obtain the initial mappings.

## Conclusions

In this article, we presented a novel approach for the mapping of sequencing reads from RNA-seq experiments. In contrast to previous approaches, ContextMap fully exploits the information on the context of a read, i.e. reads mapping in the vicinity of a read considered. Accordingly, ContextMap consists of three steps. First, the contexts are determined based on a preliminary mapping. Second, reads unmapped in the first step are aligned to the context and existing alignments are extended based on potential splice sites suggested by other alignments. In this step, all alignments satisfying the maximum mismatch criterion are taken into account for each context. Finally, the best alignment for each read is determined first for each context and then the optimal context is determined for each read.

By addressing the problem of finding the best position for each gene separately for each context, the problem size is reduced considerably. This allows evaluating a much larger number of possible alignments and accordingly positions for each read within each context. Thus, instead of considering the context only for the prediction of splice sites as done by MapSplice, we can take it into account for both complete and junction reads.

Although ContextMap can also be applied to preliminary mappings from genome or transcriptome alignments, one major application is the refinement of mappings provided by other mapping algorithms such as TopHat and MapSplice. Based on the analysis on simulated RNA-seq experiments for human and mouse, we could show that our refinement using ContextMap could improve considerably upon the accuracy of both TopHat and MapSplice predictions. In most cases, refinements of either mappings by ContextMap outperformed both original mappings. Thus, even if large fractions of reads are already correctly mapped by both approaches, mapping quality can still be improved for a substantial number of reads by evaluating the support of alternative mappings in the context of all other reads. Furthermore, if finding the optimal parameter setting is difficult as for MapSplice on the 2% error data sets, ContextMap provides a useful alternative as it obtains high accuracy even if the original mapping quality was relatively low.

## Competing interests

The authors declare that they have no competing interests.

## Authors' contributions

CCF and RZ designed the study and participated in analyzing the results. GC and TB designed ContextMap and evaluated its performance. GC implemented the ContextMap prototype. CCF drafted the manuscript. All authors were involved in revising the manuscript and read and approved the final version.
